# The Impact of Contrast Media on Lumbar Spine Bone Mineral Density Measured by Quantitative Computed Tomography

**DOI:** 10.4314/ejhs.v34i5.3

**Published:** 2024-09

**Authors:** Lahari R Shetty, Kaushik Nayak, Priyanka

**Affiliations:** 1 Department of Medical Imaging Technology, Manipal College of Health Professions, Manipal Academy of Higher Education, Manipal, Karnataka 576104, India

**Keywords:** Quantitative Computed Tomography, Contrast Media, Lumbar Spine, Bone Mineral Density

## Abstract

**Background:**

Osteoporosis is a bone disease caused by decrease in bone mineral density (BMD). Quantitative computed tomography (QCT) has proven to be an effective tool to measure the BMD of the lumbar spine. Therefore, the objective of the study is to investigate the impact of intravenous contrast media (CM) on BMD of lumbar spine measured by QCT.

**Methods:**

This is a prospective study and included a total of 141 patients (females: 71, males: 70) referred for contrast enhanced computed tomography (CECT) abdomen. First, the plain scan of abdomen was done. Contrast media was injected intravenously followed by acquisition of arterial and portovenous phase (PV) of abdomen. Plain, arterial and PV phases axial CT images were loaded on Philips BMD analysis application. A circular region of interest (ROI) measuring 30-40 mm^2^ was placed at all five lumbar vertebrae (L1-L5) and value of BMD was obtained in mg/cm^3^

**Results:**

Paired t-test was used to compare BMD in plain, arterial and PV phase. There was significant difference (p <0.05) in BMD (L1-L5) between plain (110.86±36.61 mg/cm^3^), arterial (117.04±37.95 mg/cm^3^) and PV phase (127.52±40.9 mg/cm^3^). The study also noted significant difference between males and females in BMD of lumbar spine (L1-L5) for plain and CECT abdomen (p <0.05).

**Conclusion:**

The BMD was highest for PV phase of the CECT abdomen. Therefore, the study concludes that BMD values are highly influenced by intravenous contrast media injections.

## Introduction

Osteoporosis is a systemic bone disease, which can lead to a decrease in bone strength and increases the risk of fractures. It is one of the common bone disorders in the world (1–3). It affects one in three women and one in five men after the age of fifty years (4–6). Early diagnosis of osteoporosis can decrease the risk of fracture. In the initial phases of bone loss, patients do not show symptoms. Therefore, after osteoporosis has deteriorated bones, there may be a few indications and symptoms that include a cracked or collapsing of bone causing pain, loss of height, bones shatter and stenosis (7–9). Osteoporosis is caused by a lifelong imbalance in calcium. Deficiency of calcium leads to decreased bone density and increases the risk of fractures. Dual energy x-ray absorptiometry (DEXA) scan is the gold standard imaging modality for measuring the bone mineral density (BMD) and diagnosing osteoporosis (10–13). However, BMD measurement by DEXA can be affected by technical issues such as obesity and cannot differentiate trabecular and cortical bone. DEXA has limited usage in patients with spinal abnormalities and those who had undergone spinal fusion surgeries. The accuracy of BMD measurement will be compromised in patients with osteoarthritis and spinal compression fractures (14–18).

The limitation of DEXA can be resolved by Computed Tomography (CT). Globally, a vast amount of CT scans is carried out, many of which have the potential to be utilized to provide extra information on BMD without requiring more patient time, radiation exposure, or financial outlay (19). Quantitative Computed Tomography (QCT) is one of the standard imaging modality for estimating BMD as it can measure the trabecular density. The QCT will be calibrated to the reference object of known density, it creates a three-dimensional reconstructed image and calculates the BMD (19,20).

Recent research has found that contrast media (CM) injection intravenously has an impact on BMD as measured by the CT Hounsfield unit (HU) (20). Tissue attenuation coefficients are expressed in HU and are calculated using water's relative attenuation as a reference. The HU value will be higher when the density of tissue is higher. However, the HU values are not the precise reading of bone density. As IV CM can infiltrate and augment the enhancement of bone trabecula, it is unclear if post contrast CT scans greatly exaggerate real BMD values measured using QCT. Hence, the aim of the current study is to investigate the effect of IV CM on BMD of lumbar spine estimated using QCT.

## Materials and Methods

**Study design:** This is a prospective study and approval was obtained from institutional ethic committee (IEC 2:164-2022). The study was registered under Writtenritten story-Indtaken taken written informed consent was obtained from all the study participants. The study included 141 patients with 71 females and 70 males referred for triple-phase CECT (Contrast Enhanced Computed Tomography) abdomen for various clinical indications. The triple phase of the CECT abdomen includes plain, arterial and portovenous (PV) phase phase phases. The patient's age and BMI were noted and only the patients with normal BMI were included. Patients with spinal pathology, fracture and implants were excluded from the study.

**CT Image acquisition**: All the patients had undergone CECT abdomen examination using Philips 128-slice Incisive CT. The images were acquired using standard CECT abdomen protocol. The technical parameters included: tube voltage: 120 kVp, tube current-exposure time product: 250 mAs, slice thickness and increment: 3 mm, detector width: 64 × 0.625 mm, field of view (FOV): 350 mm, matrix size: 512×512, pitch: 1.1, rotation time: 0.5s. The plain CT abdominal examination was followed by IV injection of 80ml of CM (Iohexol 300 mg Iodine /ml, GE Healthcare) and 40 ml of saline through dual head pressure injector (Guerbet, OptiVantage) with a flow rate of 4ml/s. Once the contrast enhancement in the aorta reaches this threshold of 100 – 120 Hounsfield unit (HU), the scanner automatically begins the arterial phase of imaging at post threshold delay of 8 seconds (s) followed by PV phase at post threshold delay of 45s after the start of contrast injection.

**Figure 1:**
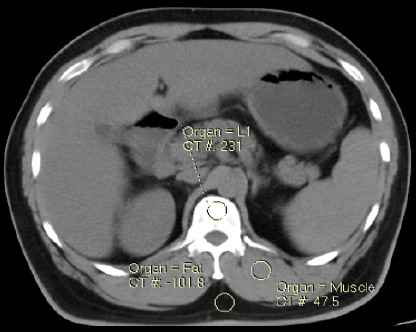
Placement of region of interest for measuring Bone mineral density

**Figure 2:**
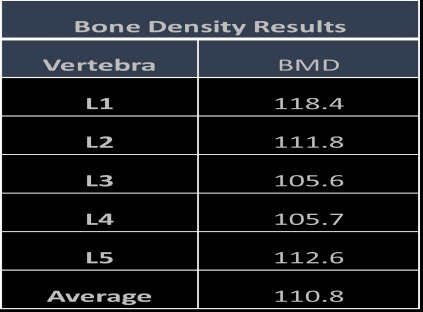
Bone density result provided by the BMD analysis application

**Measurement of BMD**: The plain, arterial and PV phase images are transferred to Philips IntelliSpace portal and images are loaded in the BMD analysis application. The default display opens withxial image. An ROI (region of interest) circular in shape measuring 30 - 40 mm^2^ was placed in central portion of the trabecular bone (L1 vertebrae). The additional ROIs will be automatically placed in the retro spinal muscle and fat tissue (figure 1). After the placement of ROI, a histogram with bell curve will be created which indicates correct ROI placement. The same steps were repeated and BMD of other lumbar vertebrae (L2 – L5) were measured. The results were obtained in the tabuand the values of BMD obtained in mg/cm3 (figure 2) were noted. All the measurements were done by two readers with more than 5 years of experience in interpreting the CT spine images and the mean values were considered as the final measurement.

**Statistical analysis**: SPSS (Statistical Package for Social Sciences) 20.0 version was used to statistically analyze the data. The mean and standard deviation of BMD of lumbar spine (L1 – L5) was calculated. ‘Paired t test’ was used to compare the BMD values between plain, arterial and PV phase. ‘One-Way analysis of variance’ was used to compare the BMD values between males and females for plain, arterial and PV phase. A p – value <0.05 was considered as statistically significant.

## Results

The study comprised of 141 patients (70 males, 71 females) with the age ranging 40-70 years (mean age = 57.57 ± 11.25). The demographic details of the patients are shown in table1. All the patients had undergone CECT Abdomen using standard CECT abdomen protocol.

**Table 1:** Demographic details of study participants

Demographic details	Male (n = 70)	Female (n =71)
Age (mean ± SD), (years)	57.77 ± 11.07	57.28 ± 11.59
Height (mean ± SD), (cm)	165.28 ± 4.83	151.87 ± 5.89
Weight (mean ± SD) (kg)	65.23 ± 3.21	56.81 ± 4.21
BMI (mean ± SD)	23.90 ± 1.83	24.61 ± 1.32

**Comparison of BMD on triple phases of CECT abdomen**: The mean and standard deviation of the BMD of the lumbar spine (L1-L5) was calculated for the plain, arterial, and PV phase of CECT abdomen and are shown in Table 2. The mean value of BMD of the lumbar spine (L1–L5) for plain, arterial, and PV phase was 110.86 ± 36.61 mg/cm^3^, 117.04±37.95 mg/cm^3^ and 127.52±40.9 mg/cm^3^ respectively. The mean BMD value of lumbar spine was higher in the PV phase compared to plain and arterial phases. There was a statistically significant difference in BMD of lumbar spine (L1-L5) between plain, arterial and PV phase (p <0.05).

**Table 2:** Mean and standard deviation (SD) of the QCT measured BMD values (mg/cm^3^) of the lumbar spine (L1-L5)

Lumbar vertebral	Plain	Arterial	PV	p value
level	(mean ±SD)	(mean ±SD)	(mean ±SD)	
L1	118.40 ±41.81	124.03 ±39.47	136.25 ±45.45	< 0.05
L2	111.77 ±40.78	119.29 ±47.01	129.46 ±50.28	< 0.05
L3	105.58 ±37.47	108.81 ±41.90	119.28 ±41.63	< 0.05
L4	105.72 ±36.51	110.63 ±38.92	125.34 ±46.25	< 0.05
L5	112.59 ±44.22	121.18 ±49.10	128.52±50.70	< 0.05
Average (L1-L5)	110.86±36.61	117.04 ±37.95	127.52±40.93	< 0.05

**Comparison of BMD between males and females**: The mean and SD of BMD in male and females for different phases of the CECT abdomen is shown in Table 3. The BMD values sa howed significant differences between males and females (p<0.05) for all the phases of CECT abdomen.

**Table 3:** Mean and standard deviation of QCT measured BMD (mg/cm^3^) for males and females

Lumbar vertebral level	Plain (mean ± SD)		Arterial (mean ± SD)		PV (mean ± SD)		p -value

	Male	Female	Male	Female	Male	Female	
**L1**	125.173 ±41.14	108.41 ±41.12	128.80 ±35.17	117.01 ±44.47	141.07 ± 43.44	129.14 ±47.76	< 0.05
**L2**	118.25 ±38.56	102.22 ± 42.40	126.66 ±47.85	108.43 ±43.94	137.96 ± 52.35	116.94 ±44.61	< 0.05
**L3**	110.77 ±34.51	97.933 ±40.56	114.02 ±39.83	101.12 ±44.00	127.61 ±40.37	106.99 ±40.74	< 0.05
**L4**	111.71 ±33.06	96.89 ± 39.74	118.64 ±34.61	98.83 ±42.12	131.34 ±46.88	116.49 ±44.23	< 0.05
**L5**	119.05 ±45.38	103.07 ±41.01	127.29 ±49.83	112.16 ±46.99	133.03 ± 45.05	121.87 ±57.83	< 0.05
**Average (L1-L5)**	117.07 ±34.87	101.72 ± 37.49	123.54 ±35.74	107.46 ±39.37	133.92 ±39.37	118.08 ±41.71	< 0.05

It was noted that the mean value of BMD was lower in females compared to males. The mean BMD value of lumbar spine (L1-L5) in males was 117.07 ± 34.87 mg/cm^3^, 123.54 ± 35.74 mg/cm^3^, 133.92 ± 39.37 mg/cm^3^ for plain, arterial and PV phase respectively. The mean BMD value of lumbar spine (L1-L5) in females was 101.72 ± 37.49 mg/cm^3^, 107.46 ± 39.37 mg/cm^3^and 118.08 ± 41.71 mg/cm^3^ for plain, arterial, and PV phase, respectively.

## Discussion

QCT is the screening tool for identifying patients with osteoporosis. As per the literature, BMD of lumbar spine was calculated using attenuation (HU) values and no studies have included all the levels of lumbar vertebrae (21–25). However, in the current study we have calculated the BMD values of lumbar spine (L1-5) using QCT before and after IV injection of CM.

In the current study, there was significant difference in QCT measured BMD values after the IV injection of CM. The mean BMD value of L1-L5 was higher in the PV phase (127.52 ± 40.93 mg/cm^3^), followed by arterial (117.04 ± 37.95 mg/cm^3^) and plain (110.86±36.61 mg/cm^3^). Similar findings were reported by other studies in which BMD of lumbar vertebrae measured using attenuation values was highest in PV phase compared to unenhanced abdomen (21, 22). However, the attenuation values are not the precise values of BMD. The study by Pompe et al (22) showed BMD difference of 19 HU and 16 HU for L1 vertebrae between plain and PV phase for non-malignant and malignant group respectively. In the study by Islamian et al. (21) and Elsayed et al (23) the BMD of lumbar vertebrae (L1-L3) in terms of attenuation was higher for enhanced phase compared to the unenhanced phase and these effects decrease with age. Jackle et al. calculated the BMD of thoracolumbar spine using QCT and they found that there was significant difference in BMD before (122.92 ± 48.16 mg/cm^3^) and after (143.80 ± 46.40mg/cm^3^) the IV injection of contrast media (25).

There was significant difference in BMD values between males and females with lower value in females compared to males in the present study. The mean BMD values for unenhanced CT was 117.0 ± 34.87 mg/cm^3^ for males and 101.72 ± 37.49 mg/cm^3^ for females. Similarly, there was significant difference in BMD values between males and females for arterial phases (males: 123.54 ± 35.74 mg/cm^3^, females: 107.46 ± 39.37 mg/cm^3^) and PV phase (males: 133.92 ± 39.37 mg/cm^3^, females: 118.08 ± 41.71 mg/cm^3^). Similar findings were observed by Islamian et al. (21), and Elsayed et al (23). in which attenuation value of L1-L3 vertebrae in enhanced and unenhanced phase was higher in males compared to females.

The study has few limitations. We did not categorize the patients based on the age and body weight as the sample size was limited and hence further studies can be done to investigate the influence of intravenous contrast on BMD of spine based on body weight and age.

In conclusion, BMD value of lumbar spine can be affected by contrast media injections and the values were highest for the porto venous phase. The study highlights the need for standardization of CT imaging protocol as a screening method for osteoporosis.
